# Characteristics and risk factors for spontaneous and postoperative consecutive exotropia in children with esotropia

**DOI:** 10.3389/fped.2023.1186666

**Published:** 2023-06-22

**Authors:** Jing Wen, Ruiying Li, Ruoshi Li, Xiaoqing Li, Dehai Zhu

**Affiliations:** ^1^ Department of Pediatric Ophthalmology, Peking University First Hospital, Beijing, China; ^2^ Peking University Children Vison Institute, Peking University, Beijing, China

**Keywords:** consecutive exotropia, follow-up period, vertical deviation, binocular function, risk factors

## Abstract

**Background:**

To investigate the risk factors for the development of consecutive exotropia (CXT) by comparing patients with spontaneous or postoperative CXT during follow-up with another group of patients who had no deviation or less than 10 prism dioptre (PD) esotropia.

**Methods:**

In this retrospective cohort study, 6 patients with spontaneous CXT (group A), 13 patients with postoperative CXT (group B), and 39 patients with no exotropia (group C) were enrolled. Probable risk factors for CXT were evaluated among the groups. Kruskal-Wallis H test was used to determine if any significant differences were present among the groups. Fisher’s exact test or Mann-Whitney U test was used for univariate analyses to compare differences between case groups or between case and control groups. The Bonferroni method was used to conduct multiple comparisons.

**Results:**

The follow-up period of spontaneous CXT patients was significantly longer than that of postoperative CXT and nonconsecutive exotropia patients (*p* = 0.035 and *p* < 0.001, respectively). The interval between alignment and CXT onset in spontaneous CXT patients was slightly longer than that in postoperative CXT patients, but not significantly difference (6.50 vs. 5.00 years, *p* = 0.072). Vertical deviation was associated with a high risk of postoperative CXT (*p* = 0.001). Most [38 (97.44%)] nonconsecutive exotropia patients had fusion; conversely, the absence of fusion function (*p* < 0.001) as well as stereoacuity (*p* = 0.029) were associated with a high risk of CXT.

**Conclusion:**

Vertical deviation and poor binocular function are strongly associated with a high risk of CXT. Children with spontaneous CXT are highly recommended to be followed-up long-term, while they maintain long-term ocular alignment before developing consecutive exotropia from comitant esotropia (CE).

## 1. Introduction

Consecutive exotropia (CXT) is an exodeviation that occurs in patients with a previous history of esotropia. The prevalence of CXT has been reported to range from 4% to 27% (1), but it is uncommon in clinical practice. It typically occurs after strabismus surgery for esotropia but can rarely occur spontaneously. Khaouam and Jampolsky (2) identified 12 cases of spontaneous CXT (0.4%) among 3,000 consecutive cases of strabismus. Romanchuk et al. (3) found that 7% of patients had spontaneous CXT in a cohort of 233 patients with a minimum of +5.00 dioptre spheres (DS) in the least hypermetropic eye. In previous studies, the prevalence of CXT increased with increased follow-up time; exodeviation can occur long-term after esodeviation treatment (1, 4).

Some studies have analysed CXT patients’ development to identify the possible risk factors for this disease. Suggested risk factors for postoperative CXT include an excessive amount of surgery, postoperative limitations of adduction, brain abnormalities, absence of fusion, amblyopia, and vertical incomitance (1, 5–7). Similar characteristic factors have also been linked with the development of spontaneous CXT (5, 8), including high hypermetropia, amblyopia, and absence of fusion. However, the mechanisms underlying the evolution towards exotropia are unclear. Because of the low prevalence of this condition, no previous reports have compared spontaneous with postoperative cases and identified the risk factors for CXT. The purpose of this study was to identify, in a cohort of CXT patients who were followed-up long-term, the risk factors for the development of CXT by comparing patients with spontaneous or postoperative CXT during follow-up with patients who had no deviation or less than 10 prism dioptre (PD) esotropia at the last visit.

## 2. Materials and methods

This study was conducted in accordance with the Declaration of Helsinki and was approved by the Ethics Committee of Peking University First Hospital (PKUFH 2022-537). Written informed consent was waived because of the use of retrospective data.

We retrospectively reviewed the medical records of patients who underwent surgery for consecutive exotropia from January 2019 to December 2021 and matched a group of patients who underwent surgery for comitant esotropia (CE) from January 2016 to January 2019. The inclusion criteria of the CXT group were as follows: consecutive exotropia, no previous strabismus surgery or only one previous CE surgery, and a follow-up period from CE onset. The inclusion criteria of the CE group were as follows: comitant esotropia, no previous strabismus surgery, and no deviation or less than 10 PD esotropia in a follow-up period of at least 2 years. The exclusion criteria were as follows: paralytic esotropia, ocular lesions, central nervous system defects, neurological or developmental disorders, and previous ophthalmic surgery.

We allocated the patients with spontaneous consecutive exotropia into group A, and the patients with postoperative consecutive exotropia into group B. The group C was composed of patients who had esotropia surgery during the same time frame as group B and did not develop consecutive exotropia.

Clinical data, including birth history, demographic characteristics, visual acuity, refraction, binocular vision and therapeutic measurements, were analysed. All patients underwent a full ophthalmological examination. Best corrected visual acuity was measured with a Snellen chart or Lea symbols chart depending on the age and level of cooperation. For comparison purposes and statistical analyses, acuity thresholds were converted to logMAR values. Amblyopia was defined as an interocular visual acuity difference of two lines or more. Instillation of 1% atropine was performed three times daily for 3 days prior to the day of cycloplegic refraction examination. The spherical equivalents (spherical error plus half the cylindrical component, SE) of the absolute refractive errors of both eyes were recorded. Hyperopia was considered significant when the degree of refractive error was >= 4.00 dioptre (D). Anisometropia was defined as a difference of 1.5 D or greater in refractive error between eyes. Prism and alternate cover tests or Krimsky tests were performed to measure the deviation at near and distance (33 cm and 6 m, respectively). Ocular motility was assessed by the same ophthalmologist (D.H.Z.). Binocular functions were assessed using the Worth four-dot test and Titmus stereotest (Stereo Optical, Chicago, IL, USA). A stereoacuity of 400 s of arc or better was defined as good.

Data analysis was performed by SPSS 23.0 (SPSS, Inc., Chicago, IL, USA). Continuous data are presented as median (range), and categorical data are presented as counts. Risk factors contributing to the development of consecutive exotropia were analysed. Kruskal-Wallis H test was used to determine if any significant differences were present among the groups. Fisher exact test or Mann-Whitney U test was used for univariate analyses to compare differences between case groups or between case and control groups. The Bonferroni method was used to conduct multiple comparisons. *P* values < 0.05 were considered to indicate statistical significance.

## 3. Results

Six patients with spontaneous consecutive exotropia (group A), 13 patients with postoperative consecutive exotropia (group B), and 39 patients with no exotropia (group C) were enrolled in this study. To determine the risk factors for spontaneous and postoperative CXT, we first aimed to determine any differences in the clinical characteristics of the patients. The patient demographic characteristics are described in Table 1. No statistically significant differences were found among the three groups in terms of sex, age, age at onset of esotropia, or birth history.

**Table 1:** Patient demographics and baseline data (*n* = 58).

Feature	Group A	Group B	Group C	*p* value
Patients (*n*)	6	13	39	—
Sex (male/female)	4/2	6/7	24/15	0.596^a^
Age [median(range), years]	12.5 (11.0∼18.0)	12.0 (7.8∼18.5)	10.1 (8.2∼18.0)	0.068^b^
Age at onset of CE [median(range), years]	3.0 (1.0∼3.0)	1.0 (0.17∼4.0)	2.0 (0.17∼5.0)	0.163^b^
Birth history [*n* (%)]	2 (33.33)	3 (23.08)	5 (12.82)	0.38^a^

Group A included patients with spontaneous consecutive exotropia. Group B included patients with postoperative consecutive exotropia. Group C included patients with no exotropia. *N,* number of patients; CE, comitant esotropia.

^a^ Fisher exact test.

^b^ Kruskal-Wallis *H* test.

Table 2 shows the surgical characteristics of these three groups. There was no significant difference in the preoperative deviation before CE surgery, type of strabismus surgery, duration from onset to CE surgery, or follow-up period between the two groups of children who had maintained orthophoria (group C) and developed CXT after CE surgery (group B). There was no significant difference in preoperative deviation (both at near and at distance) between the two CXT groups, while group A had a longer follow-up time (*p* = 0.035) and slightly longer orthostatic time (*p* = 0.072) than group B.

**Table 2:** Surgical features of the patient groups (*n* = 58).

Feature	Group A	Group B	Group C	*p* value
	(*n* = 6)	(*n* = 13)	(*n* = 39)	
Preoperative deviation before CE surgery				
At near [median(range), PD]	—	37.50 (20∼55)	30.00 (15∼60)	0.253^b^
At distance [median(range), PD]	—	37.50 (15∼50)	25.00 (15∼60)	0.163^b^
Type of strabismus surgery				
Symmetrical surgery [*n* (%)]	—	3 (23.08)	18 (46.15)	0.198^a^
Asymmetrical surgery [*n* (%)]	—	10 (76.92)	21 (53.85)	
Duration from onset to CE surgery [median(range), years]	—	2.67 (0.50∼9.00)	3.25 (0.32∼9.10)	0.512^b^
Preoperative deviation before CXT surgery				
At near [median(range), PD]	−22.50 (−40∼0)	−35.00 (−60∼−10)	—	0.210^b^
At distance [median(range), PD]	−25.00 (−30∼−20)	−30.00 (−50∼−10)	—	0.579^b^
Interval between alignment and CXT onset [median (range), years]	6.50 (5.0∼10.5)	5.00 (2.0∼10.5)	—	0.072^b^
Follow-up period [median (range), years]	10.50 (8.0∼13.0)	6.12 (3.0∼10.8)	4.68 (3.5∼6.4)	<0.001^c^^*^
				Ga vs. Gb 0.035*
				Ga vs. Gc <0.001*
				Gb vs. Gc 0.233

Group A (Ga): patients with spontaneous consecutive exotropia. Group B (Gb): patients with postoperative consecutive exotropia. Group C (Gc): patients with no exotropia. *N*, number of patients; CE, comitant esotropia; PD, prism dioptre.

* Significant difference.

^a^ Fisher exact test.

^b^ Mann-Whitney *U* test.

^c^ Kruskal-Wallis *H* test.

### 3.1. Timeframe of CXT onset

**Figure 1:**
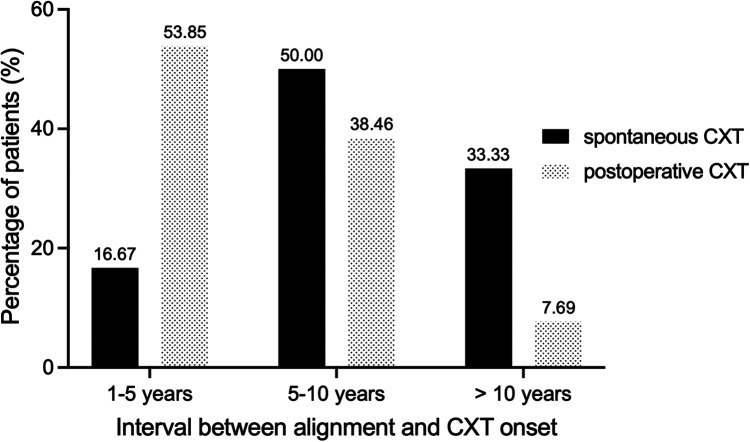
Distribution of patients according to the interval between alignment and CXT onset. Shown is the percentage of patients for each time frame. CXT, consecutive exotropia.

The peaks of incidence for spontaneous and postoperative CXT appeared in our study at 5–10 years and at 1–5 years, respectively (Figure 1). The median interval between alignment and consecutive exotropia onset in group A (6.50 years) was slightly longer than that in group B (5.00 years) (*p* = 0.072, nonsignificant). Patients in group A had a longer alignment time in the longer follow-up period (10.50 years, *p* < 0.001).

### 3.2. Factors significantly associated with CXT

The presence of vertical deviation and binocular function were significantly associated with the onset of CXT (Table 3). Three (50.00%) patients in group A, 9 (69.23%) patients in group B, and 6 (15.38%) patients in group C had vertical deviation; there was a significant difference between groups B and C (*p* = 0.001). Fusion was observed in 2 (33.33%) patients in group A, 6 (46.15%) patients in group B, and 38 (97.44%) patients in group C; most patients in group C had fusion, and this proportion was significantly different from that of groups A and B (both *p* < 0.001). Good stereoacuity was defined as 400 s of arc or better and was observed in 2 (33.33%) patients in group A, 3 (23.08%) patients in group B, and 25 (64.10%) patients in group C. The percentage in group C was higher, although not significantly, than that in groups A and B (*p* = 0.597 and *p* = 0.066, respectively). The incidence of hyperopia in group A (33.33%) was higher than that in group B and C, but not significantly difference (*p* = 0.055).

**Table 3:** Risk factors for the patient groups (*n* = 58).

Feature	Group A	Group B	Group C	*p* value
	(*n* = 6)	(*n* = 13)	(*n* = 39)	
Refractive errors				
Spherical equivalent				
OD [median(range), D]	+1.00 (−2.00∼+4.25)	+0.88 (−3.50∼+2.63)	−0.50 (−6.13∼+6.75)	0.282^b^
OS [median(range), D]	+2.00 (+0.88∼+4.00)	+0.50 (−2.25∼+3.38)	+0.25 (−6.38∼+6.38)	0.056^b^
Hyperopia >=+4D [*n* (%)]	2 (33.33)	0 (0)	2 (5.13)	0.055^a^
Anisometropia [*n* (%)]	3 (50.00)	3 (23.08)	5 (12.82)	0.074^a^
Amblyopia [*n* (%)]	1 (16.67)	1 (7.69)	4 (10.26)	0.815^a^
Vertical deviation [*n* (%)]	3 (50.00)	9 (69.23)	6 (15.38)	0.001^a^^*^
				Ga vs. Gb >0.999
				Ga vs. Gc 0.252
				Gb vs. Gc 0.001*
Fusion [*n* (%)]	2 (33.33)	6 (46.15)	38 (97.44)	<0.001^a^^*^
				Ga vs. Gb >0.999
				Ga vs. Gc <0.001*
				Gb vs. Gc <0.001*
Stereoacuity				
≤400 s/arc [*n* (%)]	2 (33.33)	3 (23.08)	25 (64.10)	0.029^a^^*^
				Ga vs. Gb >0.999
				Ga vs. Gc 0.597
				Gb vs. Gc 0.066

Group A (Ga): patients with spontaneous consecutive exotropia. Group B (Gb): patients with postoperative consecutive exotropia. Group C (Gc): patients with no exotropia. *N*, number of patients; OD, oculus dexter; OS, oculus sinister; D, dioptre.

* Significant difference.

^a^ Fisher exact test.

^b^ Kruskal-Wallis *H* test.

### 3.3. Factors nonsignificantly associated with CXT

No significant observable postoperative limitations in extraocular movements were noted in any of the postoperative CXT patients. No statistically significant differences were found among the 3 groups in terms of the presence of anisometropia, amblyopia, or refractive error. Specifically, few patients had amblyopia, and the differences in its incidence among the 3 groups were not statistically significant (*p* = 0.815).

## 4. Discussion

This study identified some risk factors for the development of consecutive exotropia in a cohort of long-term follow-up patients. Our cohort of 58 patients suggested the importance of these characteristics, such as the presence of vertical deviation, lack of fusion, and absence of stereoacuity. Although a few prior observational studies have investigated spontaneous and postoperative CXT, to our knowledge, this is the first study to analyse the clinical features of CXT and compare them with those of controls.

In our study, 3 patients (50.00%) and 9 patients (69.23%) had vertical deviation in the spontaneous and postoperative CXT group, respectively, compared to 6 patients (15.38%) in the nonexotropia control group. In previous studies, vertical deviation, including dissociated vertical deviation (DVD) and oblique dysfunction, was identified as a risk factor in spontaneous and postoperative CXT. Han et al. (9) found that DVD was a risk factor for the development of CXT after esotropia-correcting surgery with an odds ratio of 5.26. They also reported that the presence of DVD was higher in patients with spontaneous CXT than in those in the nonexodeviation group (10). They ultimately suggested that DVD might trigger the development of CXT as a result of loss of macular and peripheral fusion and imperfect binocularity. Ceylan et al. (11) reported that 26.7% of the CXT group showed inferior oblique muscle overaction compared to 7.4% of the nonexotropic group. Oblique dysfunction leads to orthotropic instability and is not beneficial for long-term alignment, which may even accelerate the development of misalignment (12). Our results highlighted the strong influence of vertical deviation in affecting long-term alignment.

Several studies have considered a high degree of hyperopia a risk factor for the spontaneous development of CXT (13, 14). Patients with high hypermetropia develop spontaneous CXT due to reduced accommodative convergence (15). According to the study of Bryselbout et al. (16), hyperopia of 4.00 D or more was reported in 55% of postoperative CXT patients and was an independent factor related to the occurrence of CXT, although not significantly (*p* = 0.05). In the present study, the median SE refractive error in the spontaneous CXT group was +1.00 D in the right eyes and +2.00 D of the left eyes. There were no significant differences in the SE refractive error between the CXT group and the control group. The proportion of patients with hyperopia at least +4.00 D was higher in the spontaneous CXT group, but the difference was not statistically significant. Additionally, only a small number of patients, 2 in the spontaneous CXT group and 2 in the nonexotropix group, had a high degree of hyperopia, which may have influenced the results. Further studies with more patients are needed to better evaluate this claim.

As other studies have illustrated, binocular function promotes alignment stability after surgical treatment or refractive correction in the long term (15, 17–19), and better binocular function translates into better eye position stability and less CXT (6, 13, 14). In this study, poor binocular function was a significant factor in the development of CXT. Long-term follow-up is highly recommended for CE patients whose fusion function has not recovered.

Our results show that the spontaneous CXT patients had a preoperative angle of deviation similar to that of postoperative patients, even with a much longer follow-up period. The interval between alignment and CXT onset in spontaneous CXT was longer than that in postoperative CXT patients. Many previous studies have suggested that the use of spectacles with reduced hyperopic correction may be considered a primary treatment for spontaneous CXT and may effectively correct exodeviation without the need for surgery (8, 10). In previous studies, 33%–98% of CXT patients showed complete resolution by reducing their hyperopic correction (4, 10, 15, 20). In this study, all 6 spontaneous CXT patients underwent strabismus surgery; additionally, they all chose hyperopic correction reduction as the first treatment and were able to maintain normal long-term ocular motility. This suggests that reducing hyperopic correction can delay strabismus surgery in spontaneous CXT patients, and they may need long-term follow-up.

This study does have several limitations. First, this was a retrospective study, and selection bias might have occurred. Because of the low prevalence, a group small sample size of patients was included in this study and the results had a high risk of a type II error. We need more data and further investigation. Second, this retrospective study was limited by incomplete data: we used the clinical distance–near relationship in the clinic and did not record the calculated accommodative convergence to accommodation (AC/A) ratio. Therefore, the role of the AC/A ratio in decompensation could not be evaluated. Third, we did not evaluate the patients for motor fusion. Further prospective studies are needed to investigate the association of the AC/A ratio and the effect of motor fusion on the results. Finally, the follow-up period was not uniform across all patients.

## 5. Conclusions

Exodeviation in patients using common treatments for CE can be observed, even though the patients showed successful ocular alignment after surgical treatment or refractive correction. This study shows that vertical deviation and poor binocular function are strongly associated with a high risk of CXT. Long-term follow-up is highly recommended for CE patients whose fusion function has not recovered after successful surgical alignment or full-correction spectacles. Children with spontaneous CXT maintain long-term ocular alignment, and their strabismus surgery may be appropriately delayed.

## Data Availability

The raw data supporting the conclusions of this article will be made available by the authors, without undue reservation.
